# The biological function of antibodies induced by the RTS,S/AS01 malaria vaccine candidate is determined by their fine specificity

**DOI:** 10.1186/s12936-016-1348-9

**Published:** 2016-05-31

**Authors:** Sidhartha Chaudhury, Christian F. Ockenhouse, Jason A. Regules, Sheetij Dutta, Anders Wallqvist, Erik Jongert, Norman C. Waters, Franck Lemiale, Elke Bergmann-Leitner

**Affiliations:** Biotechnology High Performance Computing Software Applications Institute, Telemedicine and Advanced Technology Research Center, U.S. Army Medical Research and Materiel Command, Fort Detrick, MD USA; PATH Malaria Vaccine Initiative, Washington, DC USA; Department of Clinical Research, United States Army Medical Research Institute of Infectious Diseases, Ft. Detrick, MD USA; Malaria Vaccine Branch, U.S. Military Malaria Research Program, Walter Reed Army Institute of Research, 503 Robert Grant Ave, 3W53, Silver Spring, MD 20910 USA; GSK Vaccine, Rixensart, Belgium

**Keywords:** Malaria, Antibody, Phagocytosis, Epitope, Protection

## Abstract

**Background:**

Recent vaccine studies have shown that the magnitude of an antibody response is often insufficient to explain efficacy, suggesting that characteristics regarding the quality of the antibody response, such as its fine specificity and functional activity, may play a major role in protection. Previous studies of the lead malaria vaccine candidate, RTS,S, have shown that circumsporozoite protein (CSP)-specific antibodies and CD4^+^ T cell responses are associated with protection, however the role of fine specificity and biological function of CSP-specific antibodies remains to be elucidated. Here, the relationship between fine specificity, opsonization-dependent phagocytic activity and protection in RTS,S-induced antibodies is explored.

**Methods:**

A new method for measuring the phagocytic activity mediated by CSP-specific antibodies in THP-1 cells is presented and applied to samples from a recently completed phase 2 RTS,S/AS01 clinical trial. The fine specificity of the antibody response was assessed using ELISA against three antigen constructs of CSP: the central repeat region, the C-terminal domain and the full-length protein. A multi-parameter analysis of phagocytic activity and fine-specificity data was carried out to identify potential correlates of protection in RTS,S.

**Results:**

Results from the newly developed assay revealed that serum samples from RTS,S recipients displayed a wide range of robust and repeatable phagocytic activity. Phagocytic activity was correlated with full-length CSP and C-terminal specific antibody titres, but not to repeat region antibody titres, suggesting that phagocytic activity is primarily driven by C-terminal antibodies. Although no significant difference in overall phagocytic activity was observed with respect to protection, phagocytic activity expressed as ‘opsonization index’, a relative measure that normalizes phagocytic activity with CS antibody titres, was found to be significantly lower in protected subjects than non-protected subjects.

**Conclusions:**

Opsonization index was identified as a surrogate marker of protection induced by the RTS,S/AS01 vaccine and determined how antibody fine specificity is linked to opsonization activity. These findings suggest that the role of opsonization in protection in the RTS,S vaccine may be more complex than previously thought, and demonstrate how integrating multiple immune measures can provide insight into underlying mechanisms of immunity and protection.

**Electronic supplementary material:**

The online version of this article (doi:10.1186/s12936-016-1348-9) contains supplementary material, which is available to authorized users.

## Background

Immunity to the pre-erythrocytic stage of *Plasmodium* is considered the most promising target for malaria vaccine development. The resulting protection is typically sterile, i.e., it prevents blood-stage infection and, thus, the onset of symptoms and blocks transmission of the parasites to other individuals. Most sporozoites egress from the skin into either the lymphatics or the blood stream after being injected into the skin by the mosquito during a blood meal (reviewed in [1]). The main target of anti-sporozoite antibodies is the circumsporozoite protein (CSP), which is the most abundantly expressed protein on the surface of the sporozoite. CSP has been the leading vaccine antigen for decades, albeit with variable success depending on the vaccine platform [2–5]. RTS,S/AS01, currently the lead recombinant vaccine candidate against malaria, is based on a pseudoparticle consisting of the hepatitis B surface antigen and a large fragment of the CSP, namely the central repeat region and the C-terminus of the protein. While only a few correlates of protection are known for most of the human vaccines (reviewed in [6]), it is becoming increasingly apparent that antibodies to the repeat region in RTS,S are associated with protection against malaria [7]. Whether or not they are only surrogate markers or true correlates of protection remains to be determined, and the mechanisms by which sporozoite-specific antibodies may mediate protection is still not known. There have been significant advancements in the understanding of antibody-mediated immune functions in the last few years. Until recently, the main emphasis was placed on measuring the magnitude of an antigen-specific antibody response. This does not take into account the quality of the humoral response in the form of antibody avidity and isotype, as well as epitope specificity. Functional antibody assays can address the question whether immune complexes bind to cellular receptors and trigger phagocytosis. This process results in the uptake, degradation of antigenic/pathogenic material and subsequent antigen-presentation to adaptive immune cells [8, 9].

Although it has been shown that anti-CSP repeat region antibodies are necessary for the protection elicited by RTS,S/AS01, subsequent clinical trials have shown that the magnitude of the anti-CSP repeat region antibody response is only weakly associated with protection [7, 10–13]. One explanation for this apparent discrepancy is that the quantity of anti-CSP repeat region antibodies in an antibody response may only serve as a surrogate marker for its functional capacity to neutralize the *Plasmodium* parasite. One possible hypothesis is that protection induced by the RTS,S vaccine is mediated by opsonization and phagocytosis. The uptake of opsonized parasites by phagocytic cells can lead to several possible outcomes, including phagocytosis, destruction of the parasite, followed by antigen presentation to T lymphocytes, or phagosomal escape of the parasite, which then resides in the phagocytic cell. The latter would constitute an immune escape mechanism. Although opsonization and phagocytosis have, to date, been poorly characterized in pre-erythrocytic stage immunity, this has been studied previously for blood-stage parasites and found to be associated with natural immunity to clinical malaria [14, 15], underscoring its potential role in protection for malaria vaccines.

The aim of the present study was to directly measure antibody-mediated opsonic phagocytic activity and identify how the fine specificity of the antibody response, defined as the relative response to individual epitope regions of the CS antigen, is associated with opsonization activity and protection. Towards that end, a highly sensitive, high-throughput assay to measure the phagocytic activity mediated by antibodies was developed and applied to human sera from subjects vaccinated with the RTS,S vaccine. The flow cytometry-based assay uses antigen-coated fluorescent beads to determine the frequency and intensity of phagocytosis by immune cells. This assay represents a significant improvement over a previous attempt to quantify CSP antibody-mediated opsonization due to its increased sensitivity, which allows for the identification of distinct populations of cells based on their phagocytic activity. Furthermore, unlike previous studies that measure opsonization using fluorescently labelled parasites [15, 16], the method described here can be performed by any laboratory without the need to have access to parasites.

The present study characterizes sera from subjects immunized in a phase 2 study using the standard RTS,S/AS01-vaccination regimen (three doses in four-week intervals). The efficacy of the trial, and the basic immunological evaluation have previously been reported [10]. The first step in the analysis was to measure the ability of the RTS,S/AS01-induced antibodies to opsonize CSP-coated fluorescent beads and mediate uptake by phagocytic cells. Next, the data was stratified based on the protective status of the individuals following the controlled challenge with infectious mosquitoes. No significant difference in overall phagocytic activity with respect to protection was observed. However, relative phagocytic activity, measured as ‘opsonization index’, was found to be associated with protection—protected subjects, surprisingly, had *lower* opsonization efficiency, than non-protected subjects. An in-depth analysis of the fine specificity of RTS,S/AS01-induced antibodies suggested that it was antibodies targeting the C-terminal region of CSP, and not the repeat region, that were associated with phagocytic activity, and that antibody responses from protected individuals in this study were characterized by higher repeat-specific antibody titres and lower opsonization activity. Given the limited sample size in this study (n = 20), further research on the role of phagocytic activity on protection in the RTS,S-induced antibody response is warranted, however, this study demonstrates how the combined analysis of multiple in vitro functional assays can yield insight into the mechanisms that underlie the biological activity of vaccine-induced antibody responses.

## Methods

### Study summary

Samples were obtained from one of the vaccine cohorts of clinical study NCT01366534 [10]. The efficacy of the trial, and the basic immunological evaluation have previously been reported [7]. Sera from all 20 subjects in the cohort that was vaccinated with three doses of RTS,S/AS01 on a zero–one–two-month schedule were tested for functional activity in the phagocytosis assay. Ten of the tested 20 subjects were protected in a controlled human malaria infection challenge allowing the stratification of the data with respect to protective status. Experiments were reviewed and approved by the GSK-MVI Correlates of Protection Task Force. All study participants had previously provided consent for future use of the samples for research. Consent to publish was not required as the samples were de-identified.

### ELISA assay for CS fine specificity

The ELISA assay was performed in the Malaria Serology Laboratory (USMMRP, WRAIR Silver Spring, USA) employing full-length CSP, NANP peptide and C-terminal peptide (PF16) as plate antigens as previously described [17, 18]. ELISA titres are listed as endpoint dilution at an optical density (OD) of 1.

### Phagocytosis

Phagocytosis was assessed by measuring the uptake of CSP-coated fluorescent beads by THP-1 cells. THP-1 is a human monocytic cell line that serves as an in vitro model for phagocytosis mediated by Fc receptors and complement receptors (reviewed in [19]). THP-1 cells were cultured in 75-sq cm flasks at a density of ≤5 × 10^5^ cells/ml for not more than 30 passages (about 8 weeks). NeutrAvidin-coated fluorescent beads (1 µm size, excitation/emission = 488/530 nm, Molecular Probes, Eugene, OR, USA) were incubated with biotinylated CSP [20] at 4 °C overnight. THP-1 cells are plated at 2 × 10^5^ cells/ml in 24-well plates the day before the assay. Beads were washed with PBS + 1 % BSA (wash buffer) and aliquots incubated with serially diluted sera (1:100, 1:500, 1:2500, 1:12,500, 1:62,500) for 2 h at 4 °C and then added to the THP-1 for 45 min (37 °C). The ratio of cells to beads had been previously optimized to 100 beads per THP-1 cell. Activity observed with the pre-immune serum (1:100 dilution) was used to determine the (non-specific) background activity for each study subject. Cells were transferred to FACS tubes on ice and centrifuged for 5 min and the data were acquired on a FACSCalibur (CellQuest software, Becton–Dickinson, Mountain View, CA, USA). The level of phagocytosis was determined by applying markers to the histograms. The marker M1 measures all fluorescent cells; M2 reports cells that have taken up ≥2 beads. The frequency of cells that underwent phagocytosis (Mfreq) and the mean fluorescence intensity of cells that underwent phagocytosis (MFI), related to the number of beads taken up, for M2 cells, is reported.

### Data analysis

The opsonization titre as the serum dilution that leads to 60 % of peak opsonization activity using either the Mfreq or MFI measures is reported. At each dilution point, the Mfreq and MFI measure was recorded in the M2 condition. For each opsonization measure (Mfreq and MFI), and for each subject, a four-parameter logistic fit model was used (Eq. 1) that assumes a symmetric sigmoidal curve to fit the opsonization activity (*Ops*) *versus* serum dilution ($log[Ab]$) data and uses that fitted curve to calculate the serum dilution at 60 % maximal opsonization activity ($maxOps$), as shown in Eq. 2. For each opsonization measure, the opsonization activity of the undiluted pre-immune sample for each subject was used as background opsonization activity, and subtracted from the opsonization activity at each dilution point, prior to calculating the opsonization titres. The dilution at 60 % maximal opsonization was used because for a small number of subjects, there was sufficiently high opsonization activity at even the highest dilution that a lower opsonization activity (such as 50 % of the maximum) would be outside the range of the data. Because there are two measures for opsonization activity (Mfreq and MFI), two independent opsonization titres for each subject were calculated.

1 $$Ops = d + \frac{a - d}{{1 + \left( {\frac{log[Ab]}{c}} \right)^{b} }}$$

2 $$log\left( {Ops\,titer} \right) = - c \times \left( {\frac{0.60 \times maxOps - a}{d - 0.60 \times maxOps}} \right)^{1/b}$$

The ratio of endpoint ELISA antibody titres to two antigens was calculated as a surrogate measure of the fine specificity, or the relative antibody response between them (Eq. 3). The following fine specificity ratios were calculated: NANP:CS, PF16:CS and PF16:NANP. Finally, the relative opsonization activity of a serum sample was reported as the opsonization titre of a serum sample normalized by the ELISA titre to full-length CS, referred to as the opsonization index (Eq 4). Because there are two measures of opsonization activity (Mfreq and MFI) two opsonization indices were calculated for each subject.

3 $$PF16:NANP\, ratio = log\left( {\frac{PF16\, titer}{NANP\, titer}} \right)$$

4 $$Ops\, index = { \log }\frac{Ops\, titer}{CS\, titer}$$

### Statistics

Statistical significance was assessed using an unpaired Student’s t test, assuming unequal variances between protected and non-protected subjects. 1-factor and 2-factor analyses of variance (ANOVA) were carried out between protected and non-protected subjects. To describe correlations between measures, the square of the Pearson correlation coefficient (R^2^), and *p* value, following linear fitting, were reported. All fitting and statistical analysis was carried out using the R statistical package.

## Results

### Fine specificity of CS-specific antibody response

**Fig. 1:**
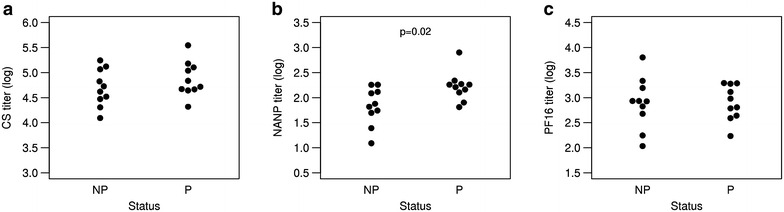
Antibody responses stratified by protection status. Endpoint ELISA antibody titres are shown for recombinant full-length CS (**a**), NANP repeat peptide (**b**), and the C-terminal PF16 peptide (**c**) for non-protected (NP) and protected (P) individuals. p value determined using Student’s t test

Previously, antibody responses to the NANP-repeat region of the CS protein in a recent clinical trial of RTS,S/AS01 was found to be significantly higher in protected subjects than in non-protected subjects [10]. In the current study, the fine specificity of the CS antibody response was characterized in terms of the response to the entire full-length CS antigen, to the C-terminal region, as well as the previously reported response to the central NANP repeat region. ELISA assays were carried out using recombinant full-length CS [20] and the C-terminus-representing PF16 peptide [18] (Fig. 1), along with the results for the NANP peptide. While, in accordance with previous findings, the magnitude of the antibody response to the NANP repeat region was associated with protection, no significant difference between the antibody response to full-length CSP or to the C-terminal region was found between protected and non-protected subjects. This suggests that the fine specificity of the antibody response, and not just its magnitude, is associated with protection.

**Fig. 2:**
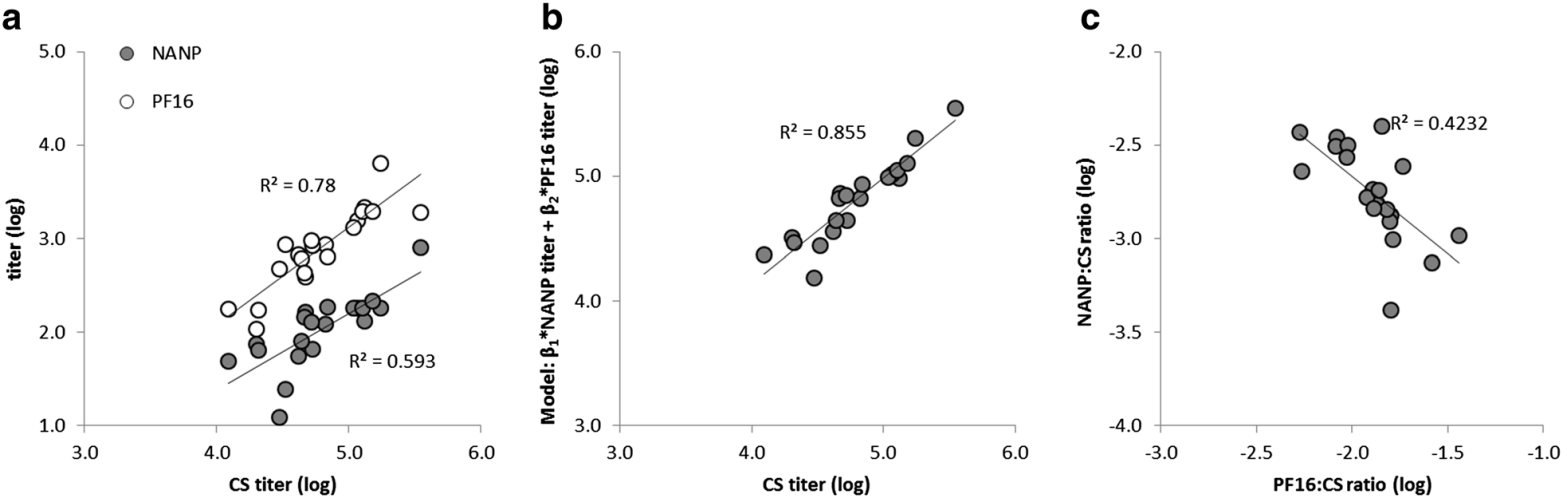
CSP antibody epitope fine specificity. **a** Endpoint ELISA antibody titres to NANP and PF16 peptides compared against ELISA antibody titres to recombinant full-length CS antigen (CS). **b** Additive model of CS antibody titres as a linear combination of NANP and PF16 ELISA antibody titres compared to ELISA antibody titres to CS. The following model was fitted using linear regression: CS titre = β_1_ × NANP titre + β_2_ × PF16 titre, and obtained values of β_1_ = 20.5 and β_2_ = 389. **c** Relative antibody responses shown as NANP:CS antibody titre ratio and PF16:CS antibody titre ratio. R^2^ values from a Pearson correlation are shown if the statistically significance is at a cut-off of *p* < 0.01

Next, the relationship between antibodies specific for distinct regions of CSP, the central repeat (NANP) and the C-terminus (represented by the PF16 peptide) *versus* the full-length protein was investigated. As expected, a correlation between the antibody titres to the NANP repeat and PF16 peptide with the full-length CSP titres was observed, demonstrating that subjects with higher antibody responses to CSP also had higher responses to the N and C-terminal regions of the antigen (Fig. 2a). Given that the NANP repeat region and the C-terminus (PF16 peptide) represent distinct antigenic regions of the CSP with little overlap, the degree to which antibody responses to these respective regions fulfilled the condition of *additivity*, that the antibody concentration specific to a full-length antigen is the sum of the antibody concentrations to antigens representing its component parts, was evaluated. The condition of additivity in antibody responses is defined in greater detail in Additional file 1. In short, antibody responses to component antigens (such as NANP and PF16) are additive, if the antibody response to the full-length antigen (CS), in terms of end-point ELISA titre, can be modelled as a linear combination of the antibody titres to the component antigens. Likewise, if CS titres cannot be modelled using NANP and PF16 titres, that means the condition of additivity is not met. Reasons for why additivity may not be observed include: (1) antibodies bind to more than one component simultaneously; (2) competition between antibodies of different epitopes; and, (3) epitopes in the full-length antigen that are not present in the component antigens.

Using linear regression, the following model was fit: CS titre = β_1_ × NANP titre + β_2_ × PF16 titre, and obtained values of β_1_ = 20.5 and β_2_ = 389. The model CS titres showed strong agreement with the experimental CS titres (Fig. 2b), suggesting that the condition of additivity between NANP titres, PF16 titres, and CS titres is met. The most straightforward interpretation of this result is that (1) the epitope regions corresponding to the NANP and PF16 antigens capture the CS response in its entirety; and, (2) CS antibodies bind to either the NANP repeat region or the C-terminal region, but not both. Indeed, an inverse relationship was found between the relative response to the NANP peptide and the PF16 peptide, represented by NANP:CS and PF16:CS titre ratios (Fig. 2c) that was statistically significant (p < 0.01), consistent with the theory that CS response can be described as the sum of the NANP and PF16 responses. Furthermore, the observed additive relationship between the repeat region and C-terminal antibody responses suggests that the NANP and PF16 peptides present the relevant epitopes, in the appropriate conformation, as they are found in the full-length CS antigen.

### Opsonization activity

**Fig. 3:**
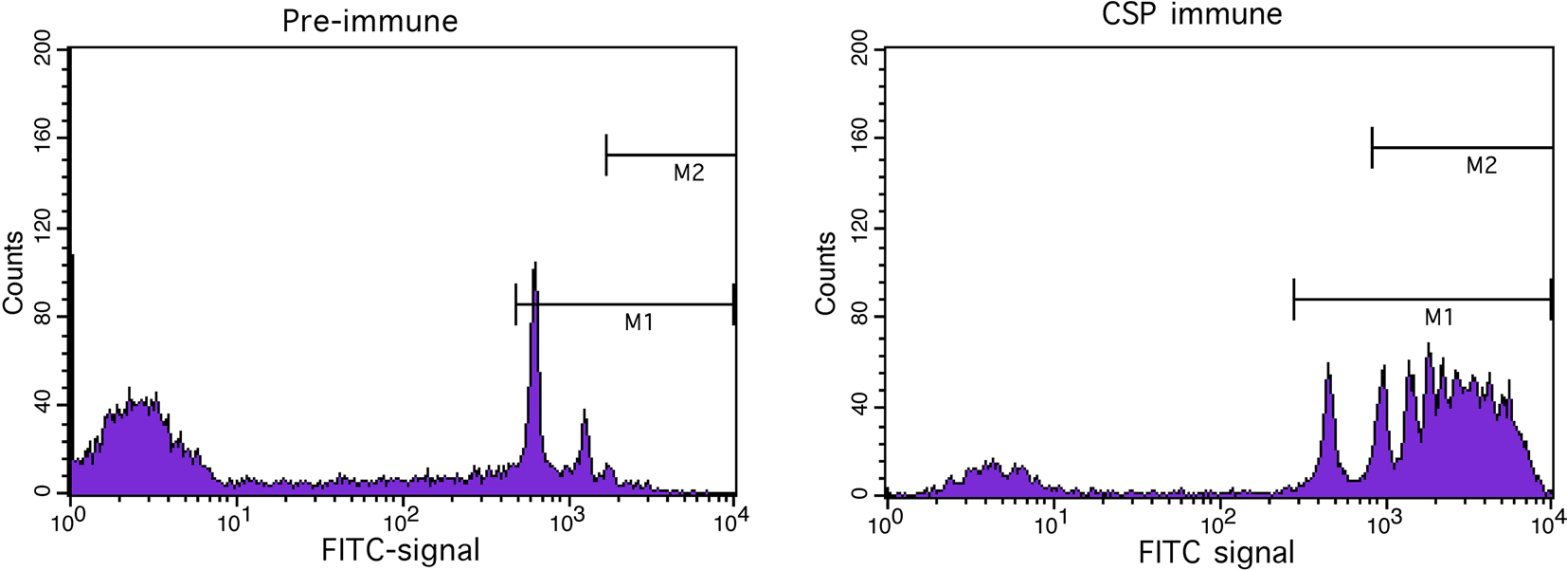
Flow cytometry to measure antibody-mediated phagocytosis in THP-1 cells. Example histograms of THP-1 cells incubated with CSP-coated NeutrAvidin beads. Markers capture either cells that have taken up at least one bead and thus are fluorescent positive (M1) or cells that have taken up two or more beads (M2). The control is a serum pool from malaria naïve US donors, CSP-specific is a serum pool that is used as positive control by the Malaria Serology laboratory at WRAIR

Given the lack of a well-defined correlate of protection based on antibody titres alone, the functional activity of the antibody response was characterized in terms of its opsonization ability using an assay that measures the antibody-mediated phagocytosis of CSP-coated fluorescent beads by the human monocytic THP-1 cell line. Following a 2-h incubation with serial dilutions of a serum sample, the CSP-coated beads were incubated with THP-1 cells for 45 min. Flow cytometry of the THP-1 cells following incubation with CSP-coated beads shows three distinct populations: cells that have not carried out phagocytosis (M0), cells that have phagocytosed one bead (M1), and cells that have phagocytosed two or more beads (M2). The level of phagocytosis for each sample was defined as the number of M2 cells (Mfreq), and the mean fluorescence intensity of M2 cells (MFI). Mfreq and MFI measures from M2 cells were used to ensure that the opsonization results reflected the most phagocytically active cells, but overall, a high correlation was found between Mfreq and MFI measures determined from M1 and M2 cells (R^2^ of 0.85 for Mfreq and 0.89 for MFI, see Additional file 2). Figure 3 shows representative data from a control (no antibodies) and CSP-specific antibody samples at a single serum dilution. It is important to note that the histogram shows clear peaks for M0, M1 and M2 populations demonstrating that the assay is capable of detecting distinct sub-populations of cells with respect to the number of phagocytosed beads. This represents a significant improvement over a previous method developed by Schwenk et al. [21], which was not sensitive enough to detect a distinct sub-population of phagocytosing cells, requiring them to use a user-defined cut-off for fluorescence intensity to quantify the number of cells that carried out phagocytosis.

**Fig. 4:**
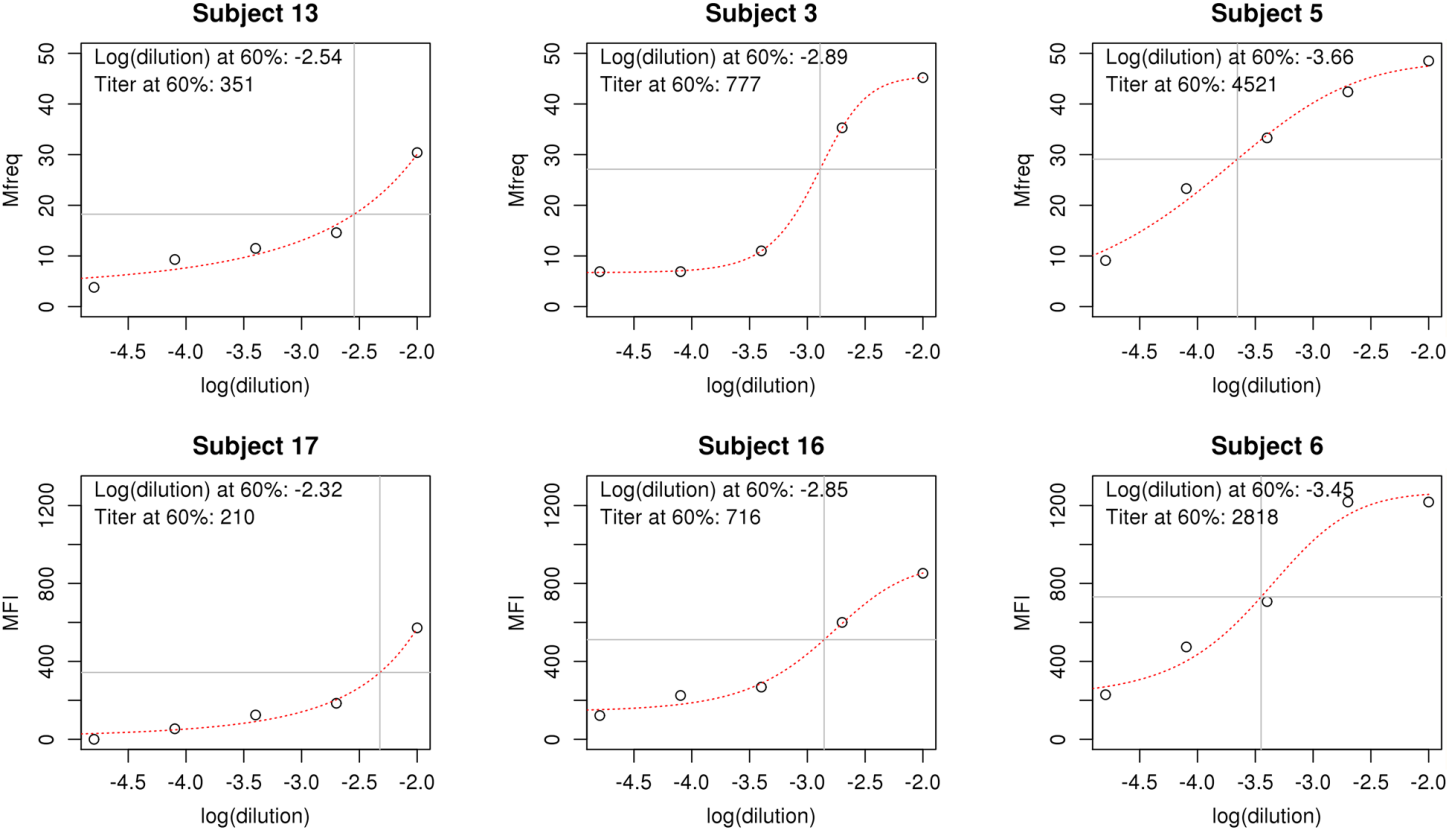
Representative opsonization activity and fitting. Representative data for opsonization activity at serial dilutions (1:100, 1:500, 1:2500, 1:12,500, 1:62,500) for two measures of opsonization: the frequency of cells undergoing phagocytosis (Mfreq, *top*) and the mean fluorescence intensity (MFI, *bottom*) of cells undergoing phagocytosis. Opsonization activity (*circles*) is shown after subtracting pre-immune serum activity. Examples of low (*left*), medium (*middle*), and high (*right*) opsonization titres are shown. The four-parameter curve fitted to the data is shown in *red*, the *horizontal line* indicates the opsonization activity at 60 % of maximum activity, and the *vertical line* indicates the log dilution corresponding to 60 % maximum activity, as determined by the fitted curve

The use of the Mfreq and MFI metrics enabled the description of phagocytosis activity in terms of (1) the frequency (the number of phagocytically active cells); and, (2) the intensity (the level of phagocytic activity of the cells, proportional to the number of CSP-coated beads taken up), respectively. Each of these four data points was measured at serial dilutions ranging from 1:100 to 1:62,500, and used these data to generate an opsonization titre for each subject that corresponds to the dilution at which 60 % of peak opsonization is observed. The availability of two data sets (Mfreq and MFI) results in calculation of two different opsonization titres. Representative data and fitted curves are shown for examples of low, medium and high opsonization titre subjects using Mfreq and MFI measures are shown in Fig. 4.

**Fig. 5:**
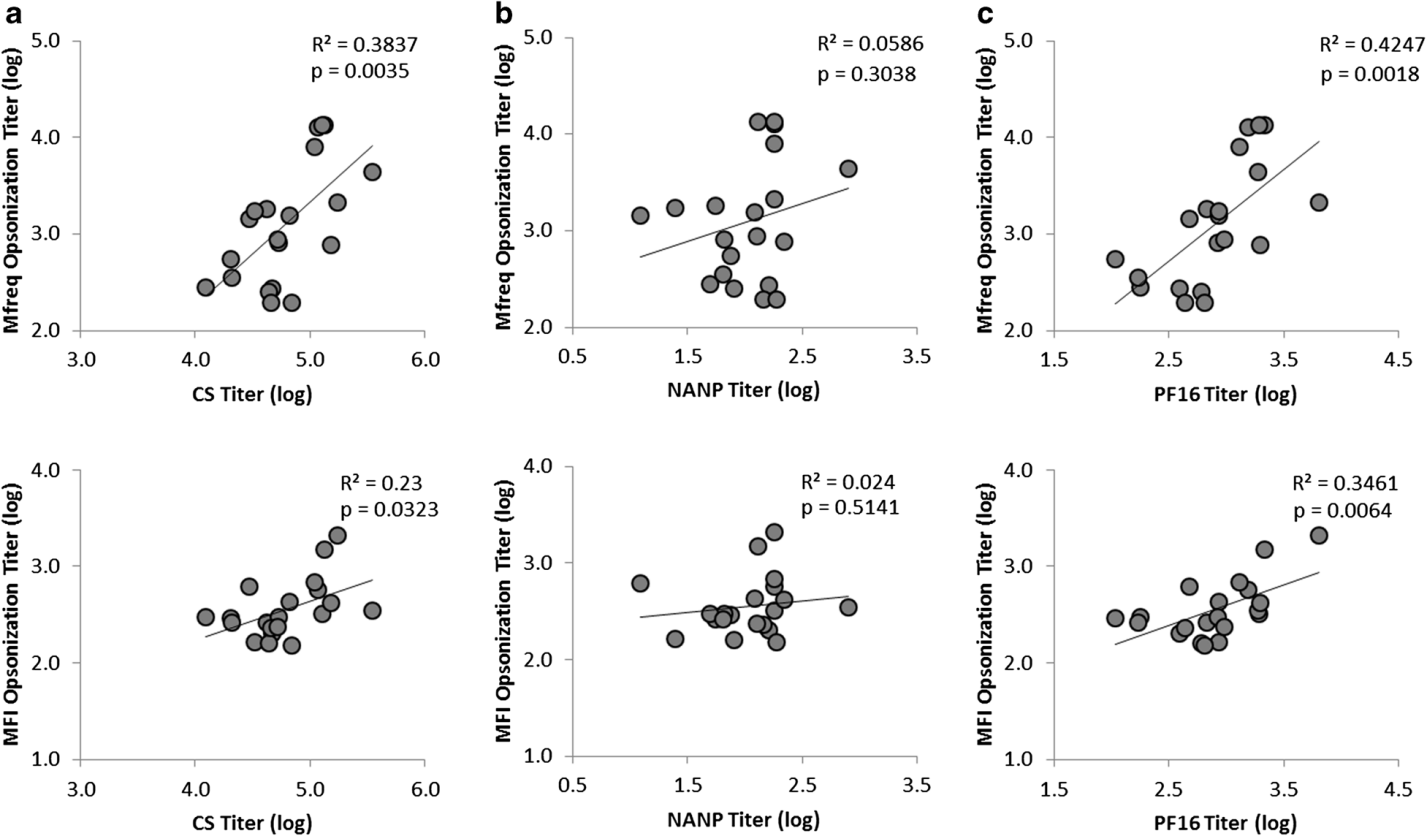
Scatterplot of opsonization titres and ELISA antibody titres. Scatterplots comparing Mfreq opsonization titre (*top*) and MFI opsonization (*bottom*) with endpoint ELISA titres for CS (**a**), NANP (**b**), and PF16 (**c**) antigens. The Pearson correlation coefficient (R^2^) and statistical significance (p) is shown

Next, the relationship between functional activity (as measured by Mfreq and MFI opsonization titres) and serological response (as measured by titres to the various regions of the CSP) (Fig. 5a) was investigated. The Mfreq opsonization titre was found to be highly correlated with anti-CSP antibody titre, but that MFI opsonization titre was not (Pearson correlation coefficients of 0.63 and 0.47), suggesting that the amount of CSP-specific antibodies in a given sample plays a much stronger role in determining the frequency of phagocytosis than the level of phagocytic activity.

### Antibody fine-specificity and opsonization activity

Given the correlation between CSP-specific antibody titres and opsonization titres, the degree to which antibody response to a particular region of the CSP antigen, such as the central NANP repeat, or the C-terminal region, was predominantly responsible for opsonization activity was explored. The opsonization titre determined by both the Mfreq and MFI was compared with the ELISA antibody titre to full-length CSP, the NANP repeat peptide, and the PF16 peptide (Fig. 5b, c).

Surprisingly, no correlation was found between the antibody titres to the NANP repeat region and opsonization titres for either the Mfreq or MFI measure. Due to the additive nature of NANP and C-terminal PF16 antibodies to the full-length anti-CSP antibody response, this finding suggested that the phagocytic activity was mediated by antibodies recognizing epitopes outside the central repeat region. A subsequent comparison between the PF16 antibody titres and both Mfreq and MFI opsonization titres showed a high correlation (Pearson correlation coefficient of 0.65 and 0.59, for Mfreq and MFI titres, respectively). This correlation was higher than that of the full-length anti-CSP response, confirming that antibodies binding to the C-terminal region of CSP are largely responsible for opsonization, and that NANP antibodies are non-opsonizing.

### Opsonization activity, fine specificity and protection

**Fig. 6:**
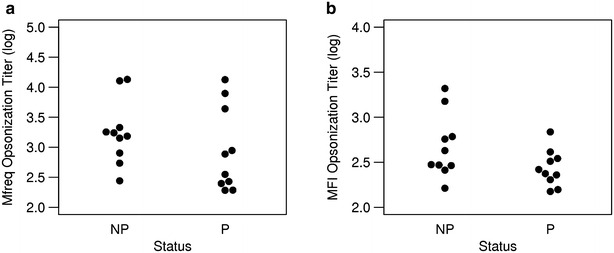
Opsonization titres separated by protection. Opsonization titres calculated from Mfreq (**a**) and MFI (**b**) opsonization activity measures is shown for non-protected (NP) and protected (P) individuals

The opsonization titres for both Mfreq and MFI were stratified based on the observed protection. Overall, there was no significant difference in opsonization titres between protected and non-protected subjects from the RTS,S vaccine study (Fig. 6). However, unexpectedly, there was a trend towards *lower* opsonization titres in protected subjects.

**Fig. 7:**
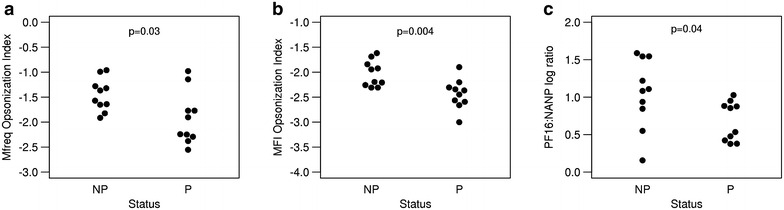
Opsonization index and epitope fine specificity separated by protection. Mfreq opsonization index (**a**), MFI opsonization index (**b**), and log ratio of PF16 antibody titres to NANP antibody titres (**c**) for non-protected (NP) and protected (P) individuals. p values determined using Student’s t test

To explore the relationship between protection, phagocytic activity and antibody titres to CSP epitopes, a measure called the opsonization index was created, which is the opsonization titre normalized by the full-length anti-CSP antibody titre. Assuming that opsonization activity of a serum sample is mediated by CSP-specific antibodies, the opsonization index measures the efficiency with which a given concentration of anti-CSP antibodies in a serum sample mediates opsonization. The opsonization index, calculated using either Mfreq or MFI, was found to be significantly lower in protected subjects than in non-protected subjects (p < 0.05 and p < 0.01, respectively) (Fig. 7a, b). This led to the unexpected conclusion that protected individuals have lower relative opsonization activities than non-protected individuals.

Since the fine-specificity analysis suggested opsonization activity of anti-CSP antibodies was primarily mediated by C-terminal antibodies and not NANP antibodies (Fig. 5), the ratio of PF16 *versus* NANP-specific antibodies of protected and non-protected subjects was compared. The results revealed that protected individuals had a lower PF16:NANP antibody titre ratio than non-protected subjects (Fig. 7c). This indicated that protected individuals not only have a higher absolute NANP response, but also a high *relative* NANP response (and low relative C-terminal response), and that this NANP-biased response is linked to both protection and a lower opsonization activity. One possible explanation is that C-terminal antibodies inhibitory and/or otherwise negatively interfere with protection, another is that the fine specificity of the antibody response is a surrogate measure of an, as-of-yet, unidentified protective mechanism.

The epitope fine specificity or relative opsonization activity was analyzed to determine if it could add any value to distinguishing between protected and non-protected subjects in addition to the previously discovered correlate for NANP titres. Performing single-variable and two-variable ANOVA analysis to distinguish between protected and non-protected subjects demonstrated that including either opsonization index or PF16:NANP ratio (p value of 0.0002 and 0.002, respectively) with the NANP titre increases the statistical significance (Table 1). This indicates that the relative opsonization activity and epitope fine specificity provide *additional* value in distinguishing protected from non-protected subjects compared to using NANP titres alone.

**Table 1:** ANOVA statistics for protected vs non-protected subjects

Type	Variables	p value
1-Factor	NANP titer	0.02
1-Factor	Opsonization index	0.004
1-Factor	PF16:NANP ratio	0.04
2-Factor	Opsonization index, NANP titer	0.0002
2-Factor	PF16:NANP ratio, NANP titer	0.002

**Fig. 8:**
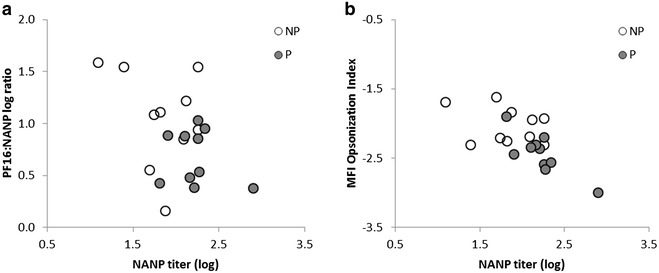
Opsonization index and epitope fine specificity combined with NANP antibody response. **a** Scatterplot of antibody fine specificity, based on PF16:NANP antibody titre ratios compared with antibody titre to NANP for non-protected (NP) and protected (P) individuals. **b** Scatterplot of MFI opsonization index and NANP antibody titre for non-protected and protected individuals. A 2-way ANOVA shows that both pairs of immune measures significantly distinguish between protected and non-protected subjects (p = 0.002 and p = 0.0002, for PF16:NANP and NANP titre, and MFI opsonization index and NANP titre, respectively)

Overall, the results suggest that not only are protected individuals characterized by higher absolute antibody titres to NANP, but that they also show systematic differences in epitope fine specificity (expressed as PF16:NANP ratio) and opsonization activity (Fig. 8). Specifically, the study reveals that protected subjects are characterized by high relative NANP titres and low relative opsonization activity.

It is possible that if a measure, such as CS titre, were positively associated with protection, then an immune measure based on its inverse (CS titre)^−1^, would be negatively associated with protection, by default. Statistical analyses were carried out to ensure that opsonization activity provides some added value in the measure of opsonization index (opsonization activity divided by CS titres) over the simple inverse measure of (CS titre)^−1^ alone. A 1-factor ANOVA using (CS titre)^−1^ showed no significant difference with respect to protection (p = 0.33) (compared to p = 0.004 for opsonization index), and a 2-factor ANOVA using (CS titre)^−1^ and NANP titre showed no added improvement from NANP titre alone (p = 0.02 for both) in distinguishing protected from non-protected individuals. This analysis shows the negative association between opsonization index and protection is not simply because it is a function of an inverse measure.

## Discussion

RTS,S/AS01 has been evaluated in several trials around the world and is on track to become the first licensed malaria vaccine in the world. As is the case for most other vaccines, no definite functional correlate of protection is known for RTS,S/AS01; most of the uncertainty related to other vaccines is associated with the contribution of cellular responses to protection [6]. In case of RTS,S/AS01-mediated protection, CD4^+^ responses and anti-CSP antibodies have both been identified as surrogate markers of protection with antibodies [7]. This provides a strong rationale for further analysing the biological activity of these vaccine-induced responses.

The present study demonstrates for the first time the relationship between antibody titres to various regions of the CSP, the contribution of epitope specific antibodies to the biological function (i.e., phagocytic activity in this study) and the underlying protective status of the samples. The current study has generated several key findings: (1) ELISA titres to the CSP central repeat region were significantly higher in protected individuals; (2) no differences in the overall ELISA titres to C-terminus or full-length CSP were detected; (3) the assessment of the opsonization titre per se did not yield a statistically significant difference between protected and non-protected individuals; (4) calculation of the opsonization index (which is a function of phagocytic activity and ELISA titres) allowed the distinction between protected and non-protected subjects; (5) unexpectedly, the opsonization index was inversely correlated with protection; (6) phagocytic activity is mediated by C-terminal antibodies and not repeat specific antibodies; (7) the predictive value of repeat specific ELISA titres or the ratio of PF16:NANP titres is lower than when using the opsonization index; and, (8) the ability to predict protection is highest when ELISA titres are combined with the opsonization index. One study has previously attempted to assess the phagocytic activity of RTS,S-induced antibodies, and although they reported a modest association between phagocytic activity and protection, their findings were limited by poor sensitivity of the assay [21]. Specifically, the low sensitivity in that study required a user-defined cut-off to be used to distinguish phagocytic from non-phagocytic cells. Beyond differences in methodology and reported opsonization measures, differences in the respective RTS,S clinical trials used in this previous study [21] and the current study, such as with immunization regimen and adjuvant conditions, preclude a direct comparison of the results.

The observation that repeat-specific antibodies were not contributing to the phagocytic activity measured in immunized subjects is a novel finding that requires further investigations into the underlying cause. The findings also indicate that a deeper understanding of the role of C-terminal antibodies in protection is needed. Studies in pre-clinical models have revealed that CSP C-terminal antibodies can play a crucial role in protection [22]. Previous work by the authors, employing a molecular adjuvant based on the complement factor C3d, demonstrated that the loss of C-terminal specificity in the overall humoral response to CSP greatly impaired protective efficacy [22]. The C-terminus of CSP contains several important functional elements, such as adhesion motifs for complement, thrombospondin and properdin. The properdin binding sequence, found in all *Plasmodium* species, may modulate susceptibility to infection [23–25]. The C-terminus has also been implicated in the initial entry of the sporozoites into hepatocytes [26] and, therefore, antibodies against this CSP-region play a role in protection [22, 27, 28]. A separate study by the authors has demonstrated that the parasites target this region of the CSP in an attempt to deviate the immune response [22], thus further supporting the hypothesis that this is a crucial region for the function and ultimately survival of the parasite. Therefore, an in-depth analysis of the biological function of C-terminal antibodies is needed before drawing conclusions regarding the overall importance in vaccine-induced protection.

The finding that phagocytosis is inversely correlated with protection was unexpected and merits further study with a larger sample size. The question remains: What is the biological consequence of the antibody-mediated uptake of the sporozoite into the phagocyte? Additional studies addressing this question are needed to determine whether *Plasmodium* employs similar immune escape strategies as has been reported for *Leishmania* (reviewed in [29]). Metacyclic promastigotes of *Leishmania* express a specialized protein, namely gp63, which quickly converts the complement factor C3b to C3bi, resulting in a preferential interaction between the opsonized promastigote and Complement receptor 3 (CR3) (rather than Complement receptor 1) [30]. The binding to CR3 still results in phagocytosis, but prevents the oxidative burst response during phagocytosis [31].

Beyond the limited sample size, there are a number of limitations of the present study in assessing the biological phagocytic activity of the RTS,S antibody response. There is mounting evidence that in vitro assays have only a limited ability to forecast the precise Fc effector mechanisms engaged by opsonizing antibodies since these assays are typically performed with monocytic cells [15, 16]. In contrast, in vivo, there is a wide range of immune cells competing with each other for the binding of immune complexes [32]. Most leukocytes express several Fc receptors and these can be classified based on their biological activity: type I receptors that provide an activating signal for the cell, while type II receptors provide a modulatory response [33]. Which Fc receptors are engaged by immune complexes is not solely governed by the isotype of the antibody, but also by the conformational state of the antibody (i.e., the glycosylation pattern). Moreover, some of these receptors are differentially expressed on immune cells, e.g., NK cells express only type I receptors and are, therefore, activated when immune complexes bind. In contrast, B cell express only type II receptors and, thus, binding of immune complexes to the Fc receptor without simultaneous ligation of the BCR results in a pro-apoptotic signal [34]. Moreover, high antibody titres and immune complexes can result in immune dysfunction by interfering with effector functions [35]. Finally, given the role of epitope density and arrangement in opsonophagocytosis, it is possible that there are differences in CSP presentation or density in sporozoites and in CSP-coated beads.

Although the current study cannot determine the ultimate biological consequences for sporozoite opsonization, it provides opsonization as a surrogate marker of protective immunity based on measuring the phagocytic activity mediated by vaccine (RTS,S)-induced antibodies, specifically antibodies targeting the CSP C-terminus. Typically, surrogate markers of protection are indicative of a protective response, but the activity measured is per se not involved in the mediation of protection [6]. Future studies may address the discrepancies between in vitro and in vivo assay systems by utilizing peripheral blood mononuclear cells of each study subject to measure the phagocytic activity within these individuals, thus accounting for the heterogeneity of phagocytic cells as well as any polymorphisms in the Fc receptors of the respective subjects.

## Conclusion

The current study demonstrates that limiting serological evaluations of vaccine studies for malaria, and likely also other infectious diseases, to the measurement of antibody titres may fail to predict vaccine efficacy unless functional assays are incorporated into the assessment. Linking the fine specificity of the humoral response with the magnitude and functional activity of the antigen-specific antibodies will have the greatest potential for success. By employing a novel phagocytosis assay, this study unveiled an unexpected inverse correlation between phagocytosis and protection against malaria mediated by RTS,S-induced antibodies.

## Additional files

10.1186/s12936-016-1348-9 Supplemental methods: Mathematical justification for additivity in antibody responses.
10.1186/s12936-016-1348-9 M1 versus M2: Scatterplot comparing M1 and M2 opsonization frequency and intensity.

## Abbreviations

CSP: circumsporozoite protein
ANOVA: analysis of variance
MFI: mean fluorescence intensity
CR3: complement receptor 3
