# Supplementary material for: The biological function of antibodies induced by the RTS,S/AS01 malaria vaccine candidate is determined by their fine specificity
Source: Malar J. 2016 May 31;15:301. doi: 10.1186/s12936-016-1348-9 (PMC4886414; doi:10.1186/s12936-016-1348-9)
Supplement: Supplementary file 1 — 10.1186/s12936-016-1348-9 Supplemental methods: Mathematical justification for additivity in antibody responses. [file 12936_2016_1348_MOESM1_ESM.docx]

**Antibody titer and concentration**

In a standard curve, the relationship between the known antibody concentration in the well ($C^{w})$ and the optical density (OD) in an ELISA assay is linear can be given by:

$$\ln\left( OD \right)=m*\ln\left( C^{w} \right)+b$$

$$\ln\left( C^{w} \right)= \frac{\ln\left( OD \right)-b}{m}$$

The parameters, $m$ and $b$ are constants that can be determined from a linear fit of the data for a given antigen. The well concentration at OD =1 ($C_{0}^{w}$) can be written as:

$$\ln\left( C_{0}^{w} \right)= \frac{-b}{m}$$

$$C_{0}^{w}=\exp\left( \frac{-b}{m} \right)$$

For a sample with an end-point ELISA titer at OD = 1 of $T$, the antibody concentration, C, is:

$$C= C_{0}^{w}*T$$

$$C=\exp\left( \frac{-b}{m} \right)*T$$

There is a linear relationship between the antibody concentration and the end-point ELISA titer:

$$C=\alpha*T$$

Where $\alpha=exp(\frac{-b}{m})$. Because the parameters of the standard curve, $b$ and $m$ are specific to the antigen and antigen plating conditions, due to factors such as antigen density, epitope presentation, etc. $\alpha$ can be described for a particular plate antigen, X, as $\alpha_{X}$:

$$\alpha_{X}=\exp\left( \frac{{-b}_{X}}{m_{X}} \right)$$

**Condition of additivity**

We can define the condition of additivity such that the antibody concentrations for a full-length antigen, X, is the sum of the antibody concentrations to $N$ non-overlapping components:

$$C_{X}= \sum_{i}^{N} C_{i}$$

Given the linear relationship between antibody concentration and titer (see above), antibody titers to the full-length antigen and to its $N$ components can be written as:

$$\alpha_{X}T_{X}= \sum_{i}^{N} \alpha_{i}T_{i}$$

$$T_{X}=\sum_{i}^{N} \frac{\alpha_{i}}{\alpha_{X}}T_{i}$$

Thus, if the condition of additivity is met, then the titer of the full-length antigen can be modeled as the linear combination of the titers of the component antigens. Conversely, if a linear combination of component antigen titers fails to capture the full-length antigen titer, that means the condition of additivity with respect to these components is not met. Reasons for why additivity may not be observed include: 1) antibodies bind to more than one component simultaneously, 2) competition between antibodies of different epitopes, and 3) epitopes in the full-length antigen that are not present in the component antigens.

$$T_{X}=\sum_{i}^{N} \beta_{i}T_{i}$$

**Model fitting to test additivity**

In the present study, we describe the antibody titers of full-length CSP as a linear combination of the antibody titers to NANP and to PF16 peptides:

$$T_{CSP}=\beta_{NANP}T_{NANP}+ \beta_{PF16}T_{PF16}$$

We used linear regression to fit values for model parameters $\beta_{NANP}$ and $\beta_{PF16}$ based on experimental values for $T_{CSP}$, $T_{NANP}$, and $T_{PF16}$.

Model parameters:

| **Name** | **Value** | **p-value** |
| --- | --- | --- |
| $\beta_{NANP}$ | 20.7 | 10^-12^ |
| $\beta_{PF16}$ | 390.3 | 10^-7^ |

The fact that CS titers can be adequately modeled using a linear combination of NANP and PF16 titers suggests that the conditions of additivity are met: NANP and PF16 peptides represent non-overlapping epitopes and, together, capture the entirety of the CS response.
