# Supplementary figures and images for: The biological function of antibodies induced by the RTS,S/AS01 malaria vaccine candidate is determined by their fine specificity
Source: Malar J. 2016 May 31;15:301. doi: 10.1186/s12936-016-1348-9 (PMC4886414; doi:10.1186/s12936-016-1348-9)

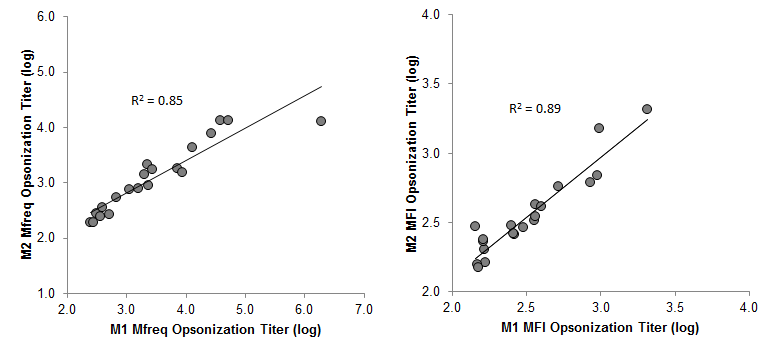

Supplement: Supplementary file 2 — 10.1186/s12936-016-1348-9 M1 versus M2: Scatterplot comparing M1 and M2 opsonization frequency and intensity. [file 12936_2016_1348_MOESM2_ESM.tif]
